# Economic valuation of farmland using natural-attribute–based indicators: A case study of Hefei, China

**DOI:** 10.1371/journal.pone.0337934

**Published:** 2025-12-30

**Authors:** Li Yuan, Xun Fan, Binrui Feng

**Affiliations:** 1 School of Agricultural and Forestry Economics and Management, Lanzhou University of Finance and Economics, Lanzhou, China; 2 School of Economics, Lanzhou University of Finance and Economics, Lanzhou, China; Gebze Teknik Universitesi, TÜRKIYE

## Abstract

The economic valuation of farmland traditionally focuses on market-based approaches, potentially undervaluing the fundamental role of natural attributes. This study aims to assess the economic value of farmland by explicitly integrating key natural attributes, including soil fertility, slope, and climatic conditions. This study develops a novel assessment framework integrating natural attributes to determine farmland economic value, with Hefei, China as the case study. The assessment combines three methods: the Comprehensive Farmland Quality Index to classify farmland by quality, the Thornthwaite-Mather model to estimate effective precipitation and water availability, and the soil productivity potential method to calculate baseline productive capacity. Together, these methods provide an objective, reproducible, and ecologically grounded basis for farmland valuation. The results reveal the economic value of farmland in Hefei. Key findings include: In 2021, the total area of farmland in Hefei was 481,500 hectares, with approximately 5% classified as first-grade land, 10% as second-grade land, 19% as third-grade land, 32% as fourth-grade land, and 33% as fifth-grade land. The estimated standard production potential per unit of farmland in 2021, from highest to lowest, was 10.28 t·hm^−2^, 9.94 t·hm^−2^, 9.31 t·hm^−2^, 8.91 t·hm^−2^, and 7.95 t·hm^−2^ for first- to fifth-grade land, respectively. Based on rice production inputs and outputs, the estimated economic value reflects a single-year, non-discounted theoretical output. The economic total value of farmland in Hefei in 2021 was calculated to be 1.363 billion yuan. Specifically, the economic value of first-grade farmland was 81 million yuan; second-grade farmland was 158 million yuan; third-grade farmland was 273 million yuan; fourth-grade farmland was 444 million yuan; and fifth-grade farmland was 407 million yuan. Among all districts of Hefei, the economic value of farmland in 2021 ranked from highest to lowest as follows: Chaohu, Feidong, Feixi, Lujiang, Shushan, Yaohai, Luyang, and Baohe.

## 1. Introduction

Farmland represents a complex system combining natural and economic dimensions. It possesses both natural and economic attributes. As a vital natural resource, the natural attributes underpin productivity, while economic attributes emerge from their utilization [1,2].

In China, valuing farmland economically is essential to advancing ecological civilization and sustainable development. However, although existing studies have examined farmland valuation through market-based approaches, there remains a critical gap in methodologies that comprehensively account for natural attributes as the foundation of economic value [3]. Current valuation methods often overlook how soil quality, topography, and climate directly influence agricultural productivity and thus economic worth [4]. Therefore, developing methods to account for the economic value of farmland from the perspective of its natural attributes is an effective approach to address this issue. It also serves as a valuable supplement to existing farmland economic assessment methods [5].

Hefei, the capital city of Anhui Province, China, is endowed with unique natural conditions due to its geographic location. The city has representative and typical soil, terrain, and climate characteristics for both Anhui Province and China as a whole. Hence, exploring methods for assessing the economic value of farmland in Hefei from the perspective of its natural attributes holds significant scientific value and practical importance. By analyzing the farmland economic value in Hefei, this study also aims to provide reference value for international farmland valuation research. As noted by Hu [3], relying solely on market transaction cases often underestimates the contribution of natural productivity. This underscores the need for approaches that integrate natural baselines.

This study contributes a novel theoretical and methodological framework for farmland economic valuation. Unlike traditional methods that rely solely on market prices or income outputs, this research emphasizes the primary role of natural attributes—such as soil fertility, climate conditions, and terrain—in shaping economic value. By integrating the Comprehensive Farmland Quality Index (CFQI), the Thornthwaite-Mather model, and the soil productivity potential estimation, the study develops an objective, reproducible, and ecologically grounded approach. This framework not only enhances the precision of spatial valuation but also supports sustainable land-use planning. It is particularly relevant for regions facing rapid urbanization and farmland degradation. Therefore, the study holds significant practical relevance for farmland protection, ecological compensation, and the advancement of ecosystem service valuation.

## 2. Literature review

As research on farmland economic value assessment has deepened, the methods for assessment have become increasingly diverse. These methods can be categorized into the following five types: (1) Market Transaction Value Method [6]: This method is based on the economic principle of supply-demand equilibrium prices and involves adjusting transaction prices from farmland market cases to determine farmland value. While it provides direct market reflections, it heavily depends on sufficient transaction samples, which are often lacking. (2) Mathematical Model Method [7,8]: This approach calculates the economic value of farmland using econometric models, taking into account various indicators that influence farmland value. It allows for rigorous multi-factor analysis but faces challenges in collecting comprehensive data. (3) Income Capitalization Method [9,10]: This method estimates farmland’s economic value based on the economic output value of annual crop yields, discounting this value to derive farmland’s economic value. It is simple and widely applied but is highly sensitive to market fluctuations and resource input constraints, often neglecting natural site factors. (4) Soil Productivity Potential Estimation Method [11]: This method calculates farmland value based on the natural productivity potential of the soil, in combination with yield benefits. It focuses solely on the natural attributes of farmland, without considering other attributes [12]. While it objectively captures natural baseline productivity and provides stable theoretical benchmarks, it does not account for farm management practices that also affect yields. (5) Subjective Willingness Evaluation Method [13]: While simple and feasible, this approach is prone to typical CVM biases such as hypothetical bias and strategic responses, making data precision harder to ensure.

It is challenging to guarantee the objectivity and stability of the assessment results because the market transaction value method, mathematical model method, income capitalization method, and subjective willingness evaluation method all carry a high degree of uncertainty and are strongly influenced by social, economic, policy, and cognitive factors. The soil productivity potential estimation method [14,15], on the other hand, reveals the value derived from the soil’s natural properties, objectively reflects the natural attributes of the land, and demonstrates year-to-year consistency under similar natural conditions. However, it should be acknowledged that this method does not incorporate variations from agricultural management practices, which can also significantly impact actual yields. It can be considered as the baseline value and theoretical value for the economic value of farmland. Thus, the soil potential estimation method is an effective approach for assessment the economic value of farmland from the perspective of its natural attributes.

In current research on farmland economic valuation, the ultimate objective of farmland quality analysis lies in establishing distinct farmland quality grades. Conventional valuation methodologies typically involve sequential procedures: first classifying farmland into quality grades, then computing economic value based on these classifications [9]. Notably, farmland quality analysis and economic valuation constitute separate research objectives, as quality metrics are not directly incorporated into quantitative value calculations. However, farmland quality is intrinsically linked to agricultural productivity potential, serving as a critical determinant of production capacity [16,17]. Omitting quality parameters in productivity potential assessments compromises analytical accuracy, thereby diminishing the precision of economic valuation outcomes. Consequently, it is imperative to develop a systematic framework that explicitly integrates the correlation between farmland quality and productivity potential, while advancing computational methodologies that couple quality grading systems with economic valuation metrics.

Current farmland economic valuation studies predominantly employ parcel-scale assessment units, with limited scholarly exploration of grid-scale valuation frameworks [18–20]. Grid-based farmland quality analysis offers enhanced spatial resolution, enabling more precise identification of spatial heterogeneity in farmland quality [21]. Gridded assessment units not only delineate nuanced quality disparities across land parcels but also facilitate quality grading at finer spatial resolutions. This granular approach ensures higher precision in quality-based area delineation, thereby effectively capturing spatial variations in farmland economic value. Such spatially explicit calculations provide robust empirical support for economic valuation while significantly improving assessment accuracy. Recent studies have further highlighted the effectiveness of using grid-based approaches to evaluate ecosystem services and farmland productivity. For example, Wu et al. applied a grid framework to assess landscape patterns and their ecosystem service values in China’s agro-pastoral transitional zones [22], while a study in Chengdu employed NDVI-based grid assessments to quantify food supply and soil conservation services [23]. These recent works demonstrate the growing adoption of grid-scale analyses for capturing spatial heterogeneity in ecosystem functions, reinforcing the relevance of this study’s methodology.

In summary, first, addressing the current research status where farmland economic valuation primarily relies on economic attributes, there is a scarcity of studies incorporating natural attributes for economic valuation. This study seeks to transcend the limitations of the prevailing research perspective that “characterizes economic value solely through economic attributes,” instead positioning natural attributes—the primary characteristics of farmland—as the prerequisite and foundation for valuation. It draws on valuation methodologies based on farmland productivity to advance economic valuation research. Second, while the soil productivity potential valuation method exclusively focuses on natural attributes for economic valuation [24], its application in this study fully demonstrates the foundational role of natural attributes in shaping farmland’s economic value. Third, existing farmland economic valuation studies predominantly employ land parcels as assessment units, lacking micro-scale resolution. This study proposes grid-based valuation to enhance computational precision. Finally, to address the current disconnect between farmland quality analysis and economic value calculation in existing valuation frameworks, this study integrates farmland quality analysis into soil productivity potential calculations to reflect the influence of natural baseline conditions on economic value. Specifically, a comprehensive farmland quality index (CFQI) is utilized to progressively adjust soil productivity potential across different quality grades, thereby deriving productivity potentials for farmlands of varying qualities. Concurrently, CFQI is applied to classify farmland areas by quality grade. The adjusted productivity potential for each quality grade is multiplied by crop yield values to calculate unit-area economic value. The economic total value is then obtained by summing the products of unit-area values and their corresponding quality-specific areas. This approach enhances the accuracy of economic valuation.

## 3. Study area and data sources

### 3.1. Overview of the study area

**Fig 1:**
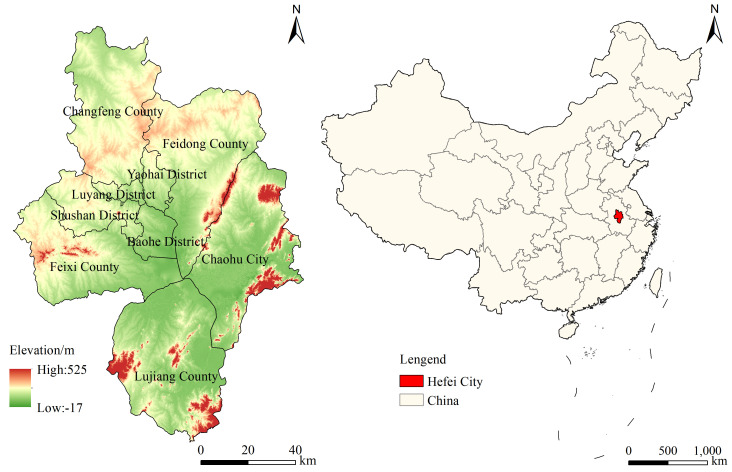
Overview of the study area.

Hefei, the capital of Anhui Province, is located between 30°56′ to 32°33′ north latitude and 116°40′ to 117°58′ east longitude, in the central part of Anhui Province(Fig 1). Hefei is a city in eastern China that is encircled by Chaohu Lake and located on the western edge of the Yangtze River Delta. Hefei’s topography is composed of three primary landforms: low-lying plains, low-mountain residual hills, and mountainous terrain. Specifically, low-lying plains account for approximately 65% of the area, low-mountain residual hills about 25%, and mountainous terrain about 10%, based on interpretation of DEM data. Located in a mid-latitude region, Hefei experiences a subtropical monsoon climate characterized by distinct monsoon influences, four distinct seasons, mild temperatures, and moderate rainfall. Hefei’s main economic crops are rapeseed and wheat, but the two main grain crops are rice and wheat.

The greatest grain reservoir in southern China is found in Anhui Province, which also serves as a major basis for agricultural output. As the capital city of Anhui Province, Hefei is located in the heart of the province and holds representative status among prefecture-level cities.

### 3.2. Data sources and processing

The data used in this study include meteorological data, land use data [25], digital elevation data, and soil attribute data. The base maps were obtained from the Ministry of Natural Resources of the People’s Republic of China (https://www.mnr.gov.cn/). The 2021 annual average temperature data were sourced from the Resource and Environment Science Data Center of the Chinese Academy of Sciences (http://www.resdc.cn/), while annual precipitation data were obtained from the Earth Resources Data Cloud Platform (www.gis5g.com). (Note: the Earth Resources Data Cloud Platform is operated by the National Geomatics Center of China, Ministry of Natural Resources).

The annual precipitation data obtained from the Earth Resources Data Cloud Platform (www.gis5g.com) are derived from interpolated meteorological station observations and remote sensing calibration products. The spatial resolution of precipitation data is 1 kilometer, and temperature data are also provided at 1-kilometer resolution. The data are provided in the WGS_1984 geographic coordinate system.

To ensure consistency with other spatial datasets, the precipitation data were reprojected to the WGS_1984_UTM_Zone_50N coordinate system, using the “Project Raster” tool in ArcGIS 10.8. All raster layers were resampled to a 30-meter resolution for spatial alignment across indicators.

The 2021 land use data for Hefei were obtained through interpretation processing of 30-meter resolution Landsat images (http://landsat.visibleearth.nasa.gov/). The digital elevation model (DEM) data were sourced from the NASA Shuttle Radar Topography Mission (SRTM) 3-arcsecond (~90-meter) dataset, available through the U.S. Geological Survey (USGS) Earth Resources Observation and Science (EROS) Center (https://www.usgs.gov/centers/eros/science/nasa-srtm3-data-dictionary). The soil attribute data were sourced from the National Earth System Science Data Center – Loess Plateau Science Data Center (http://loess.geodata.cn), the Earth Resources Data Cloud Platform (www.gis5g.com), etc.

Given the nature of the research subject, Hefei’s farmland utilization vector data were extracted from the 2021 Anhui Province land use data. Farmland slope data were obtained from 30-meter DEM data. Using ArcGIS 10.8, the Euclidean distance tool was used to calculate the distances between farmland, rural residential regions, and water systems to determine geographic parameters. A five-level buffer zone was generated with intervals of 500 meters to derive locational indicators. Qualitative indicator data were obtained by establishing these buffer zones using the buffer tool. Soil texture indicators were based on the top 0–20 cm layer, while soil organic matter indicators used the 0–100 cm layer.

To ensure consistency in the application of raster data and the precision of the results, the projection coordinate systems of Anhui Province’s meteorological raster data, digital elevation data, and certain soil attribute raster data were standardized using the “Project Raster” tool in ArcGIS. All raster data were converted to a unified coordinate system, and the raster cell sizes were standardized. All data utilized in this study were obtained from publicly available sources. To ensure temporal consistency across datasets, all data sources used in this study were temporally aligned to the 2021 benchmark, thereby providing a stable and consistent analytical foundation for the research.

All spatial analyses and mapping were performed using ArcGIS 10.8, while multi-criteria decision analyses were implemented using SuperDecisions software.

## 4. Research methods

This study selects Hefei City, Anhui Province, as the research area to establish a farmland economic valuation system centered on farmland quality grading and potential productivity assessment. Utilizing the soil productivity potential valuation method, we derive the economic total value of farmland across different quality grades, ultimately obtaining the aggregate economic value of Hefei’s farmland. The methodological framework is structured as follows:

(1) Determination of Comprehensive Farmland Quality Index (CFQI): Evaluation indicators were selected based on the farmland Quality Grading standard (GB/T 33469−2016) [26]. The CFQI was calculated using the integrated fertility index method to assess farmland quality.
(2) Classification of Farmland Areas by Quality Grade: Farmland areas were categorized into distinct quality grades using the natural breaks classification method, applied to the CFQI values.
(3) Calculation of Climatic Productivity Potential: This model was applied using Hefei’s 2021 annual average temperature and precipitation data to compute climatic productivity potential with the Thornthwaite Memorial Model [27].
(4) Determination of Farmland Productivity Potential: The output NPP was explicitly converted from grams per square meter (g/m^2^) to tons per hectare (t/hm^2^) by dividing by 10,000, ensuring consistency across all productivity calculations. Climatic productivity potential was then iteratively adjusted using CFQI to derive refined farmland productivity potential values [28].
(5) Standard Yield Estimation for Productivity Potential by Quality Grade: Based on quality grade classifications, the Zonal Statistics tool in ArcGIS was used to calculate average productivity potential values for each grade, generating standard yield estimates.
(6) Unit-Area Economic Value by Quality Grade: Market cost and revenue data for agricultural products were integrated with standard yield values. The soil productivity potential valuation method was applied to calculate unit-area economic values for each quality grade.
(7) Farmland Economic Total Value Calculation: Economic total value was aggregated using the summation method, multiplying unit-area values by their corresponding quality-specific farmland areas and summing the results. To ensure spatial consistency and methodological reproducibility, all calculations were conducted at a consistent spatial resolution (evaluation unit size of 30 m × 30m).

**Fig 2:**
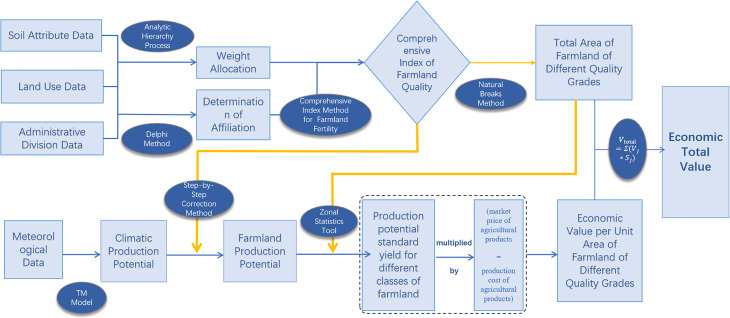
Route for accounting for the economic value of farmland.

The calculation process of the economic value of farmland is as shown in Fig 2.

### 4.1. Calculation of comprehensive farmland quality index

#### 4.1.1. Division of evaluation units and establishment of attribute database.

The evaluation unit is the most fundamental element in the process of farmland quality grade assessment and serves as the basis for subsequent farmland quality grade assessment. This study adopts the overlay method to divide the evaluation units [29]. By overlaying the current land use map, soil data map, and administrative boundary vector map of Hefei, a basic evaluation unit map is generated. An evaluation unit is composed of identical soil units, administrative division units, and land use type blocks. While there are significant differences between different evaluation units, they can still be compared. Small fragmented patches generated from the overlaying process are eliminated, resulting in the final 20,439 farmland fertility evaluation units in Hefei. The average area of these evaluation units is approximately 5.1 hectares, and they are irregular polygons rather than uniform hexagonal grids, reflecting the overlay structure of land use, soil, and administrative boundaries.

To ensure spatial consistency and methodological reproducibility, all spatial datasets—including land use, soil, and topographic data—were uniformly resampled to a spatial resolution of 30 meters using bilinear interpolation. This resolution achieves a balance between spatial accuracy and computational efficiency and is widely adopted in regional-scale land quality assessments. All spatial data were projected to a unified coordinate system (WGS_1984_UTM_Zone_50N) to ensure alignment. The overlay and resampling operations were conducted using ArcGIS 10.8, while the calculation of farmland quality indicator weights was performed using Super Decisions 3.2. The spatial data processing workflow included standardizing coordinate projections, rasterizing vector data with consistent resolution, integrating multi-source thematic layers through spatial overlay, and extracting attribute statistics using zonal analysis.

The establishment of an attribute database is primarily for the unified management of large volumes of data. In this study, attribute data are linked to evaluation unit data through common fields, which are generated using the “Zonal Statistics as Table” tool in ArcGIS 10.8, thus creating the attribute database.

#### 4.1.2. Selection of indicators and determination of weights.

(1) Selection of Indicators

With reference to relevant studies and the Farmland Quality Grading standard [26], which integrates agricultural zoning criteria and indicator systems, and considering the natural characteristics of farmland in the study area, 16 evaluation indicators across five categories-site conditions, physical properties, chemical properties, ecological factors, and locational factors-were selected based on the principles of representativeness, relevance, and complementarity [30–32].The selection of indicators including terrain position, field slope, plow layer texture, soil bulk density, gravel volume, available water content, farmland fragmentation, soil pH, soil organic matter, cation exchange capacity, organic carbon, and total nitrogen was based on the Farmland Quality Grading standard (GB/T 33469−2016). Indicators such as distance to water systems, distance to residential areas, soil water conservation capacity, and soil retention capacity were selected based on relevant literature [33]. The ecological factors (I15, I16) were informed by empirical soil and water conservation models developed in similar agro-ecological regions. The chosen indicators and their rationales are listed in Table 1.

**Table 1:** Data sources and weighting of indicators of farmland quality grades.

Target layer	Criterion layer	Indicator layer	Selection reason	Data sources	Weights
Cropland quality grade	Topographic conditions	Topographic Areas *I*_*1*_	Indicators of the geographical environment that reflect the suitable growth of crops	Earth Resources Data Cloud Platform	0.1191
		slope *I*_*2*_ (°)	Indicators of the geographical environment that reflect the suitable growth of crops	Earth Resources Data Cloud Platform	0.2381
	Physical	Cultivated texture *I*_*3*_	A physical indicator that reflects the composition of soil particles	World Soil Database	0.0632
		Soil bulk density *I*_*4*_ (kg/dm^3^)	A physical index that reflects soil tightness conditions	National Earth System Science Data Center	0.0309
		Soil gravel volume *I*_*5*_ (cm^3^/100 cm^3^)	A physical indicator that reflects the gravel content in the soil	National Earth System Science Data Center	0.0188
		Effective water content *I*_*6*_ (mm/m)	Physical indicators influencing the effects of water in the soil on plant growth and development	ISRIC Soil Data [36]	0.0442
		Farmland fragmentation *I*_*7*_ (%)	A physical index that reflects the degree of concentration and contiguity of farmland	Earth Resources Data Cloud Platform	0.0173
	Chemical properties	Soil pH *I*_*8*_	A chemical indicator that reflects the pH of the soil	National Earth System Science Data Center	0.0434
		soil organic matter *I*_*9*_ (g/kg)	A chemical indicator that reflects the fertility of the soil	Earth Resources Data Cloud Platform	0.0819
		Cation exchange capacity *I*_*10*_ (cm^3^/kg)	Chemical indicators that reflect the fertilizer retention performance of soil	National Earth System Science Data Center	0.0434
		Organic carbon *I*_*11*_ (kg/m^3^)	A chemical indicator that reflects the fertility of the soil	National Earth System Science Data Center	0.0756
		Total nitrogen *I*_*12*_ (g/kg)	A chemical indicator that reflects the fertility of the soil	National Earth System Science Data Center	0.0264
	Location factors	Distance from the water system *I*_*13*_	Degree of Reflection Irrigation Convenience	Geospatial data cloud	0.0426
		Distance from settlements *I*_*14*_	Degree of Reflection Cultivation Convenience	Earth Resources Data Cloud Platform	0.0426
	Ecological factors	Soil water conservation *I*_*15*_	It reflects the water conservation capacity of farmland ecosystem	Earth Resources Data Cloud Platform	0.0562
		Soil retention capacity *I*_*16*_	Reflects the soil conservation service capacity of farmland	Earth Resources Data Cloud Platform	0.0562

(2) Determination of Weights

The indicator weights were determined using the Analytic Hierarchy Process (AHP), a method widely used by scholars in the field of land value assessment to calculate indicator weights [34,35]. AHP is a multi-objective decision-making method used to handle a limited number of alternatives. It decomposes the elements related to the decision into different levels—goals, criteria, and alternatives—and derives the weight of each factor through pairwise comparisons, analyzing from the lowest level to the highest level. Finally, the weight of each alternative with respect to the overall goal is calculated. The hierarchical structure model for farmland quality grades was constructed according to the AHP, establishing the goal level, criteria level, and indicator level, with a hierarchical diagram illustrating the hierarchical structure and the subordinate relationships between factors.

A judgment matrix was then created for the farmland quality grade indicators. Key pairwise comparison values are summarized in Appendix A in S1 Appendix, which reflects expert judgments, for example assigning a 5 to soil fertility versus topography based on perceived relative importance. A scale from 1 to 9 and their reciprocals were used to compare the relative relevance of linked factors inside the current level for each factor in the previous level. This study utilized Super Decisions software (version 3.2) to calculate the relative weights of each indicator. The consistency ratio was 0.08, and the results passed the consistency test. The calculated indicator weights are presented in Table 1.

To enhance methodological transparency, a simplified example of the pairwise comparison process and key entries from the judgment matrix are provided in Appendix A in S1 Appendix. The comparison follows the 1–9 scale developed by Saaty, where a value of 1 indicates equal importance between two factors, and 9 indicates extreme importance of one factor over the other. For instance, in evaluating the relative importance of ‘Soil Fertility’ over ‘Topography’, a value of 5 was assigned, reflecting moderate to strong preference based on expert judgment. These comparative values formed the basis for constructing the complete judgment matrix used to derive indicator weights through the AHP.

#### 4.1.3. Determination of membership degree.

The determination of the membership degree for numerical evaluation factors in current research primarily involves using the Delphi method to assess the measured values and derive the corresponding membership degree. The results are then used to fit a numerical membership function, into which the measured values of each evaluation factor are substituted to calculate the membership degree of each factor [37,38].

Based on fuzzy mathematics theory and considering the characteristics of the selected indicators in the study area, the relationship between the numerical evaluation indicators and farmland quality in the study area is categorized into three types of membership functions: decreasing-type, peak-type, and linear-type functions [39].

For evaluation factors that fit the decreasing-type function model, larger values correspond to lower levels of farmland quality. The detrimental impact of these variables on the quality of agriculture, however, tends to level off after a while. Farmland quality is higher for evaluation criteria that fit the peak-type function model when their values are closer to a particular range. Factors that follow the linear-type function model have a linear relationship between their values and farmland quality.

In this study, slope, farmland fragmentation, and soil gravel volume are categorized under the decreasing-type function; soil bulk density and soil pH are categorized under the peak-type function; and soil organic matter content, cation exchange capacity, organic carbon content, total nitrogen content, and effective water content are categorized under the linear-type function, as shown in Table 2.

**Table 2:** Numerical Indicator affiliation functions for farmland quality grade evaluation.

The name of the metric	The type of the function	Function formulas	a-value	b-value	c-value	Lower limit value of u	Upper limit value of u
Slope	Decreasing type	Y_i_ = 1/[1 + a(u_i_-c)^2^]	0.0157	——	0	0	28.0476
Farmland fragmentation	Decreasing type	Y_i_ = 1/[1 + a(u_i_-c)^2^]	0.0282	——	0.2393	0	16.9944
Soil gravel volume	Decreasing type	Y_i_ = 1/[1 + a(u_i_-c)^2^]	0.0055	——	1.2537	5.66	19.3860
Soil bulk density	Peak type	Y_i_ = 1/[1 + a(u_i_-c)^2^]	57.0546	——	1.2597	1.21	1.5
ph	Peak type	Y_i_ = 1/[1 + a(u_i_-c)^2^]	0.0034	——	0	5.4	7.2
organic matter	Straight type	Y_i_ = au_i_ + b	0.0417	−0.343143	——	8.9648	32.2732
cation	Straight type	Y_i_ = au_i_ + b	0.0120	−1.735604	——	138.7333	228.4375
Organic carbon	Straight type	Y_i_ = au_i_ + b	0.0482	0	——	0	22.4
Total nitrogen	Straight type	Y_i_ = au_i_ + b	1.1069	−0.72424	——	0.72	1.81
Effective water content	Straight type	Y_i_ = au_i_ + b	0.0015	0	——	27.2	36.8200

Note: Y_i_ is the degree of affiliation of the i_th_ factor; u_i_ is the specific numerical value of the indicator; C_i_ is the standard indicator; a is the coefficient; b is the intercept.

The determination of data affiliation of conceptual indicators belongs to the category of conceptual affiliation functions, which are conceptual indicators whose traits are qualitative, non-numerical and have a non-linear relationship with the quality of farmland. This type of factor does not require the establishment of an affiliation function model and can be directly given the degree of affiliation using the Delphi method. In this paper, we referred to the “Farmland Quality Grade”, other academic results and the basis of expert scoring as the criteria for the classification of the conceptual indicators [34,38], as shown in Table 3.

**Table 3:** Farmland quality grade evaluation conceptual type indicator affiliation score.

The name of the metric	Conceptual qualitative indicator grading criteria									
	1	0.9	0.8	0.7	0.6	0.5	0.4	0.3	0.2	0.1
Topographic Areas	Impact Plains	Alluvial fan plains	lacustrine plains	Flood plains	Storm the mesa		Flood mesas		Small undulating mountainous terrain	
Cultivated texture	Tsubado	Clay loam soil	Silty loam	Sandy loam	clay	Heavy clay		Loamy sandy soil		Sandy soil
Distance from settlements	Closer		Close		Moderate		Far		Farther	
Distance from the water system	Closer		Close		Moderate		Far		Farther	
Soil retention capacity	Stronger		Strong ability		Moderate		Weak		Weaker	
Water conservation capacity	Stronger		Strong ability		Moderate		Weak		Weaker	

The membership function parameters (a, b, c) used in this study were calibrated based on expert consultation and reference to previous empirical studies on farmland quality assessment. The calibration followed the approach in Bijanzadeh E. & Mokarram M., which specifically addressed fuzzy membership fitting for farmland grading [40]. Specifically, the parameters were derived by combining expert judgment with regression fitting using historical farmland quality survey data from Hefei and similar agro-ecological zones.

For conceptual indicators, the Delphi method was employed to establish affiliation scores. The Delphi panel consisted of 15 experts, including 6 university researchers, 5 officials from agricultural departments, and 4 experts from local land planning institutes. A panel of 15 experts in soil science, agricultural engineering, land use planning, and rural development participated in two rounds of scoring. These experts were selected from academic institutions, government agencies, and agricultural research centers to ensure diverse and balanced perspectives.

The final scores were determined based on the consensus reached in the second round. Parameter values and affiliation scores were validated by comparing them with national farmland classification standards (GB/T 33469−2016, Ministry of Agriculture and Rural Affairs) to ensure methodological robustness.

#### 4.1.4. Determination of a composite index of farmland quality.

After calculating the weight of each indicator and the membership degree of different indicators, the cumulative method is used to calculate the comprehensive index of farmland quality [41]:


$$\text{IFI}=\Sigma ({\text{C}}_{\text{i}}*{\text{F}}_{\text{i}})$$ (1)

where: IFI is the composite index of farmland quality; C_i_ is the combined weight of the i_th_ evaluation index; F_i_ is the degree of affiliation of the i_th_ evaluation index. The index IFI ranges from 0 to 1, where higher values indicate better farmland quality. The lower bound represents very poor quality land, while the upper bound indicates optimal conditions.

### 4.2. Determination of the area of farmland of different quality grades

Farmland quality grading is classified from the perspective of agricultural production [42] by evaluating the ability of farmland to satisfy the sustained output and quality and safety of agricultural products constituted by land strength, soil health and field infrastructure through the composite index method [43]. Considering the distribution of the comprehensive index of farmland quality, it was chosen to use the natural breakpoint method. The natural break thresholds identified were: Grade I (0.6477–0.7499), Grade II (0.5738–0.6477), Grade III (0.4991–0.5738), Grade IV (0.4186–0.4991), and Grade V (0.2840–0.4186), clearly delineating the farmland quality levels. Based on the comprehensive index of farmland quality, in descending order, different quality farmland is divided into five grades of farmland quality between the highest and lowest points on the curve of the comprehensive index of farmland quality. The larger the comprehensive index of farmland quality, the higher the level of farmland quality. The smaller the comprehensive index of farmland quality, the lower the level of farmland quality. The highest level of farmland quality in the first class and the lowest level of farmland quality in the fifth class. According to the comprehensive index nodes of farmland quality, the total area S_j_ of farmland at different quality grades is obtained, with values of 1, 2, 3, 4, and 5.

The Jenks natural breaks method was selected for classification because it minimizes within-group variance while maximizing between-group variance, making it suitable for datasets with skewed or clustered distributions such as the farmland quality index. Compared with equal-interval classification, Jenks better reflects the inherent data structure and ensures that each quality grade captures statistically meaningful differences.

To assess the robustness of this classification, we conducted a sensitivity check by comparing the Jenks method with an equal-interval classification. The spatial distribution patterns and statistical characteristics (e.g., average quality index per grade) remained consistent, indicating that the use of Jenks breaks does not introduce significant bias. Therefore, the method is both technically justified and robust.

### 4.3. Measurement of climatic production potential of farmland

At the regional scale, crop production potential is mainly determined by the spatial distribution pattern of light, temperature, water, and soil in the region [44], where climatic (light, temperature, and water) production potential refers to the maximum yield that can be achieved by farmland in the study area as determined by light, temperature, and water conditions, provided that the basic soil conditions for agricultural production are fully met [45].

This study adopts the empirical method as the calculation method for the climate production potential of farmland, and the Thornthwaite Memorial model, which is more mature in the empirical method, was selected as a model to account for the potential production capacity of farmland in the study area by establishing a functional relationship between climatic factors and crop yields, whose main mechanism is to characterize crop production capacity in terms of the combination of climatic factors [46,47]. Compared to the Penman–Monteith equation and process-based NPP models, the Thornthwaite model provides a simpler empirical approach, making it well-suited for regional-scale studies with limited detailed meteorological inputs [27]. This model has been widely applied in evaluating crop productivity and vegetation potential across different climatic zones in China, particularly in regions with similar monsoon-influenced subtropical climates [42,48]. The model uses the annual actual evapotranspiration to predict production capacity, and the formula is as follows:


$$\text{L}=\text{0}.\text{05}{\text{T}}^{\text{3}}+\text{25T}+\text{300}$$ (2)

$$\text{V}=\frac{\text{1}.\text{05R}}{{\left[\text{1}+{\left(\frac{\text{1}.\text{05R}}{\text{L}}\right)}^{\text{2}}\right]}^{\frac{\text{1}}{\text{2}}}}$$ (3)

$${\text{NPP}}_{\text{T}}=\text{3000}*[\text{1}-{\text{e}}^{-\text{0}.\text{0009695}(\text{v}-\text{20})}]$$ (4)

Where: T is the mean annual temperature (°C); V is the average annual actual evapotranspiration (mm); R is the annual precipitation (mm); L is the annual maximum evapotranspiration (mm) and NPP_T_ is the climatic production potential of vegetation determined by evapotranspiration (g/m^2^); e is the base of the natural logarithm.

The use of this model utilizes the ArcGIS “grid calculator” tool to calculate the processed annual average temperature and precipitation data using the Thornthwaite Memorial model, and explicitly converted the output NPP from grams per square meter (g/m^2^) to tons per hectare (t/hm^2^) by dividing by 10000, i.e., 1 g/m^2^ = 0.01 t/hm^2^, to maintain consistency across all productivity calculations. This conversion enables consistency with the unit of productivity potential used throughout the farmland valuation framework.

### 4.4. Determination of farmland production potential

In fact, the climatic production potential at the regional scale is often constrained by other conditions, and the actual yield of farmland cannot reach the level of climatic production potential because soil conditions are hardly optimal [49,50] and soil conditions are not homogeneous in different areas within the same region. Thus, it was necessary to adjust the climatic production potential using a correction that accounts for spatial soil fertility differences. Therefore, this paper used the stepwise correction method to correct the climate production potential based on the previously obtained farmland quality grade index, and obtained the corrected unit farmland production potential MPP [14] (t/hm^2^) in the study area. The formulation employs a direct product (IFI×NPPT), which assumes a proportional (linear) adjustment of climate-limited productivity by the integrated fertility level. This is justified because the CFQI or IFI mainly captures soil and site properties, acting as a multiplicative attenuation factor on climatic potential. While more complex nonlinear interactions could exist in reality, the linear formulation offers a clear, interpretable adjustment and is widely used in regional productivity assessments [14]. Formular as follows:


$$\text{MPP}=\text{IFI}*{\text{NPP}}_{\text{T}}$$ (5)

Where: MPP (Modified Production Potential) is the modified farmland production potential; NPP_T_ is the vegetation climate production potential determined by evapotranspiration; IFI is the Integrated Fertility Index (IFI) of farmland quality.

### 4.5. Calculation of standard yield for different grades of farmland

Farmland of the same quality grade in the region has very similar soil condition limitations, therefore, crop yields of farmland of the same quality grade are also roughly equivalent. Based on the classification of farmland quality grades, the Zonal Statistics tool in ArcGIS was used to partition productivity potential values by grade and calculate their averages, thereby obtaining the standard yields for each quality grade. These standard yields serve as the benchmark for the production potential of each grade, denoted as MPP_j_.

### 4.6. Assessment of unit-area economic value for farmland by quality grade

The economic value of farmland is defined as the cumulative return of a piece of farmland over a period of time [18].The economic value assessment in this paper adopts the soil potential valuation method, which considers soil productivity to be determined by the size of the soil’s potential crop yield.

Under the condition of dividing farmland into different quality grades, the representative yield standard MPP_j_ of each quality class of farmland is calculated, and the standard yield of crops in different classes of farmland is used to find out the income and get the economic value of the unit of farmland in different grades.

The agricultural product revenue is based on the double-cropping rice system (spring and summer seasons) in Hefei City. By evaluating seasonal variations in yield, production costs, and market prices, the actual economic benefits of rice cultivation were reflected. Specifically, the average rice market price (p) was determined to be 2,640 yuan per ton and the average production cost (c) was 1,760 yuan per ton, derived from the *Anhui Provincial Agricultural Statistical Yearbook* (2021) and Hefei local agricultural cost-benefit bulletins. The farmland economic value was calculated as the product of farmland productivity potential (yield per unit area) and agricultural product revenue (value per unit area), thereby establishing a linkage between natural attribute value and market value and converting production capacity into economic output [48]. The calculation formula is shown in Equation (6):


$${\text{V}}_{\text{j}}={\text{MPP}}_{\text{j}}*(\text{p}-\text{c})$$ (6)

In the formula where: p is the market price of agricultural products; c is the production cost of agricultural products; MPP_j_ (Modified Production Potential) is a representative yield standard (t/hm^2^) for different levels of farmland; V_j_ is the economic value of the j_th_ grade farmland.

The values of *p* and *c* were derived from the *Hefei Statistical Yearbook (2021)* and agricultural cost-benefit data published by the Hefei Municipal Bureau of Agriculture. Specifically, the average rice market price (*p*) was calculated as 2,640 Chinese yuan per ton, while the average production cost (*c*) was estimated as 1,760 Chinese yuan per ton, based on regional field surveys and published government statistics.

Given the double-cropping rice system (early and late season), seasonal variations in *p* and *c* were accounted for. Separate values of price and cost were collected for spring and summer rice, and the annual average values were computed as the arithmetic mean of the two cropping seasons. This averaging method ensures that the final economic value per unit area reflects the overall annual production cycle.

### 4.7. Economic total value assessment

Multiply the unit economic value of farmland of different grades by the total area of farmland of different grades to obtain the economic total value:


$${\text{V}}_{\text{total}}=\Sigma ({\text{V}}_{\text{j}}*{\text{S}}_{\text{j}})$$ (7)

Where: V_total_ is the economic total value; V_j_ is the economic value of the j_th_ class of farmland; S_j_ is the area of the j_th_ class of farmland, and j is taken as 1, 2, 3, 4, 5. This aggregation was performed by first calculating the economic value per evaluation unit according to its quality class and then summing across all evaluation units. This approach ensures spatially explicit summation and minimizes rounding errors compared to direct area-level aggregation.

## 5. Results

### 5.1. Farmland quality classification results

Based on the calculated comprehensive index of farmland quality (IFI), five farmland quality grades were classified using the natural breakpoint method. In accordance with the farmland Quality Grading national standard (GB/T 33469−2016), specifically its provisions on farmland quality grading indicators for the middle and lower reaches of the Yangtze River, the farmland quality grading results for Hefei City were validated through field verification and expert review. The results demonstrated a high degree of alignment with actual conditions, thereby ensuring the scientific rigor and reliability of the classification outcomes. The CFQI thresholds for each grade were: Class I (0.6477–0.7499), Class II (0.6078–0.6477), Class III (0.5773–0.6078), Class IV (0.5327–0.5773), and Class V (0.3962–0.5327), corresponding to increasing limitations in soil fertility and topography. The classification is shown in Table 4.

**Table 4:** Classification of Farmland Quality Grades Based on CFQI and Their Spatial Characteristics in Hefei.

	First class	Second class	Third class and other places	Fourth-class and other places	Fifth place
Composite index of farmland quality	0.6477 ~ 0.7499	0.6078 ~ 0.6477	0.5773 ~ 0.6078	0.5327 ~ 0.5773	0.3962 ~ 0.5327
Area(hm^2^)	24692.89	49650.41	91483.20	155614.00	160033.49

To enhance the interpretability of the classification, each farmland quality grade was assigned a descriptive label based on dominant landscape, soil fertility, and accessibility characteristics. For example, Class I (0.6477–0.7499) represents the most fertile plains with organic matter often above 2%, Class II (0.6078–0.6477) corresponds to slightly less fertile plains or low hills, and so on, with declining fertility and increasing terrain constraints through Class V. First-class farmland areas are typically fertile alluvial plains with high productivity and good irrigation access (e.g., central Changfeng County). Second-class areas are slightly less fertile plains or low hills with moderate irrigation (e.g., Feidong). Third-class areas are mixed terrain with moderate soil quality and accessibility. Fourth-class farmland includes hilly and transitional zones with shallow soils and more fragmented plots (e.g., eastern Chaohu and Lujiang). Fifth-class areas are predominantly mountainous or peripheral zones with poor accessibility, lower fertility, and limited water resources (e.g., Feixi and southern Chaohu). These labels are summarized in Table 4.

Based on the classification of farmland quality levels according to the comprehensive index, the area statistics for farmland of different quality levels were calculated, as shown in Table 4, yielding the following results:

In 2021, Hefei had a total farmland area of 481,400 hectares. The distribution of farmland area across different quality levels is as follows: First-grade farmland covers 24,700 hectares, representing approximately 5% of the total farmland area. Second-grade farmland covers 49,700 hectares, representing approximately 10% of the total farmland area. Third-grade farmland covers 91,500 hectares, representing approximately 19% of the total farmland area. Fourth-grade farmland covers 155,600 hectares, representing approximately 32% of the total farmland area. Fifth-grade farmland covers 160,000 hectares, representing approximately 33% of the total farmland area.

Analyzing the distribution across different administrative regions: In Baohe District, fifth-grade farmland occupies the largest proportion, representing about 63% of the district’s total farmland area. In Chaohu City, fifth-grade farmland occupies the largest proportion, representing about 42% of the city’s total farmland area. In Feidong County, fifth-grade farmland occupies the largest proportion, representing about 52% of the county’s total farmland area. In Feixi County, fifth-grade farmland occupies the largest proportion, representing about 55% of the county’s total farmland area. In Lujiang County, fourth-grade farmland occupies the largest proportion, representing about 49% of the county’s total farmland area. In Luyang District, second-grade farmland occupies the largest proportion, representing about 33% of the district’s total farmland area. In Shushan District, fourth-grade farmland occupies the largest proportion, representing about 75% of the district’s total farmland area. In Yaohai District, fourth-grade farmland occupies the largest proportion, representing about 43% of the district’s total farmland area. In Changfeng County, fourth-grade farmland occupies the largest proportion, representing about 36% of the county’s total farmland area.

The specific results are rounded to two decimal places, as shown in Table 5.

**Table 5:** Farmland Area by Quality Grade Across Administrative Regions (hm^2^).

County(District)	First grade	Second grade	Third grade	Fourth grade	Fifth grade	Total
Baohe	45.41	323.23	653.02	340.55	2306.83	3669.04
Chaohu	2493.1	4134.98	15500.8	18479.64	29128.09	69736.6
Feidong	3685.99	19874.53	12608.84	17571.45	58758.1	112499
Feixi	62.51	1880.58	13654.03	18285.95	42121.82	76004.9
Lujiang	1589.93	8896.8	16834.84	50363.67	24121.19	101806
Luyang	98.61	870.89	800.67	367.54	511.37	2649.08
Shushan	26.49	627.8	3853.98	16257.79	941.65	21707.7
Yaohai	7.27	232.07	1245.03	1721.62	825.23	4031.21
Changfeng	16682.9	12809.54	26331.97	32225.8	1319.2	89369.4
Total	24692.2	49650.41	91483.2	155614	160033.49	481473

**Fig 3:**
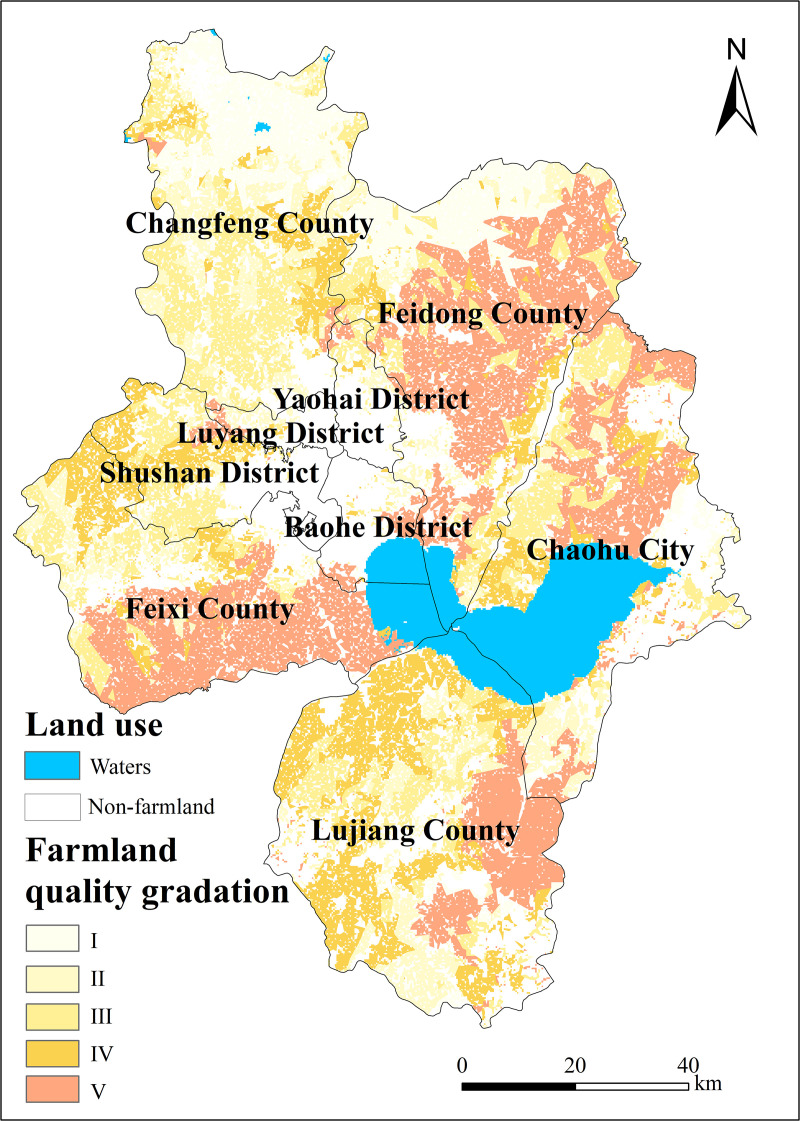
Classification of farmland quality in Hefei City.

From the distribution of farmland across different grades, the overall quality of farmland exhibits a trend of being more prevalent in the west and less in the east, as well as more concentrated in the north and less in the south. First-class farmland is the least abundant, predominantly located in Changfeng County; the number of second-class farmland is relatively small, mainly in Feidong and Changfeng Counties; third-class land is being mainly distributed in Chaohu, Feidong, Feixi, Lujiang, Shushan, and Changfeng; fourth-class farmland is more numerous, mainly in Chaohu, Lujiang, and Changfeng; and fifth-class farmland is the most numerous, mainly in Chaohu, Feidong, Feixi, and Changfeng. As illustrated in Fig 3.

### 5.2. Standard output of production potential of farmland at different levels

**Fig 4:**
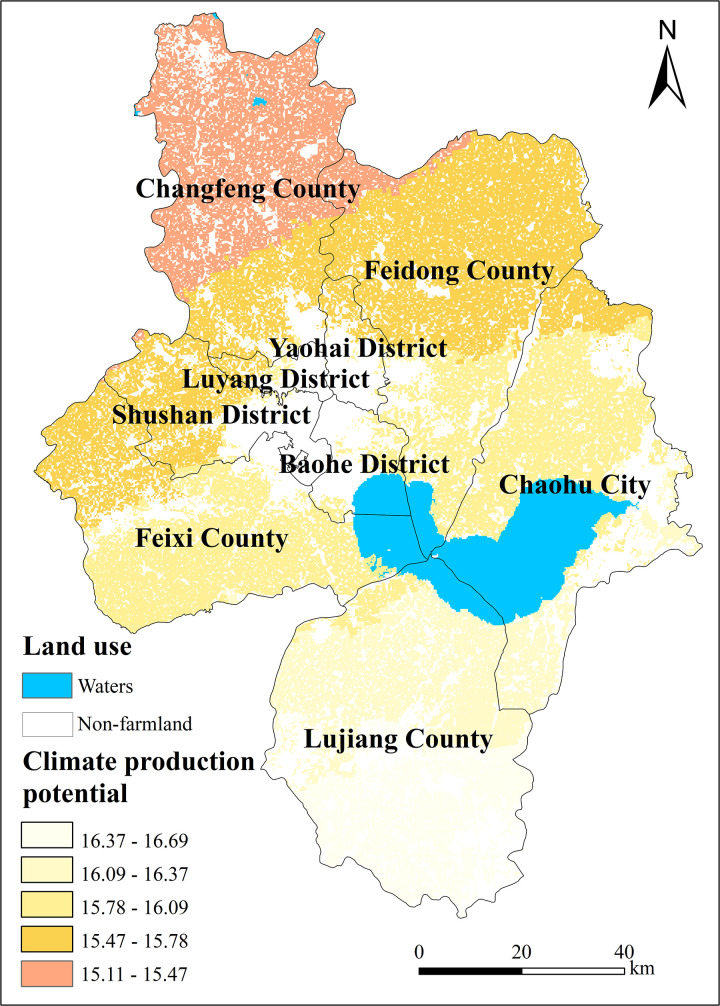
Classification of climate production potential in Hefei City.

The analysis revealed that the climate production potential of Hefei City in 2021 ranged from 15.11 to 16.69 t/hm^2^. Generally, this potential exhibited a trend of being higher in the south and lower in the north. Specifically, it was higher in the southern districts of Feixi and Lujiang and lower in the northern part of Baohe, capturing the main directional pattern. In the higher latitude areas of the study region, a decreasing trend was observed from northeast to southwest, as illustrated in Fig 4.

**Fig 5:**
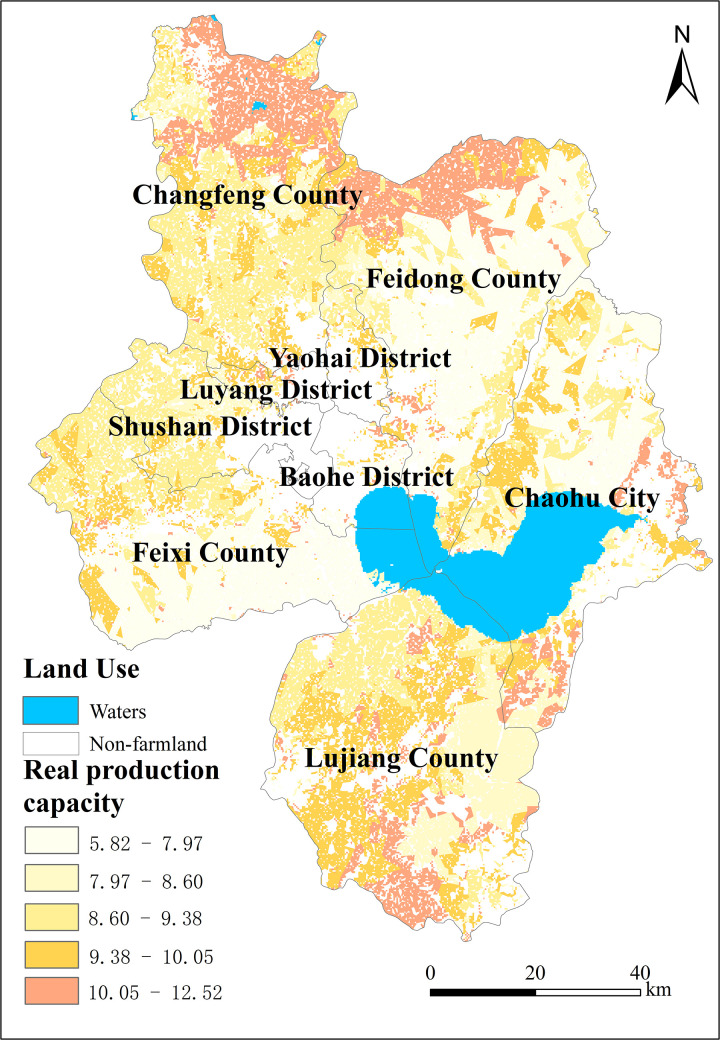
Classification of farmland production potential in Hefei City after revision.

In 2021, the calculated production potential of farmland in Hefei City ranged from 5.82 to 12.52 t/hm^2^. This value was adjusted based on the comprehensive index of farmland quality, taking into account the climate production potential. Overall, the production potential displays a trend of being higher in the west and lower in the east, as well as higher in the north and lower in the south, as shown in Fig 5. This spatial gradient reflects the combined influence of topographic, soil, and irrigation conditions. Areas with higher production potential, typically found in Changfeng and Lujiang counties, coincide with flat terrain, better irrigation, and fertile soils. In contrast, lower potential zones are mainly distributed in the eastern hills and southern mountainous edges of Feixi and Chaohu, characterized by shallow soils and limited water availability.

To enhance visual clarity, Fig 5 uses a diverging color scheme with a light-to-dark gradient, where darker shades represent higher productivity values and lighter tones indicate lower values. This intuitive color ramp helps clearly delineate production potential gradients across the study area.

**Fig 6:**
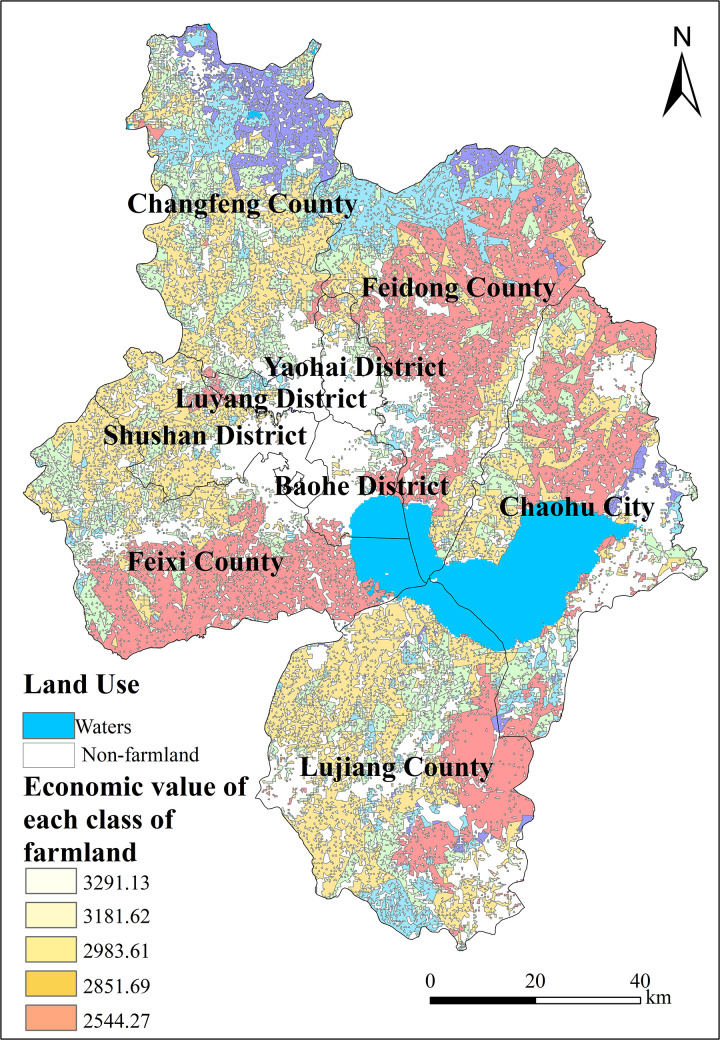
Economic value standards corresponding to the fifth grade quality farmland in Hefei City.

Therefore, the production potential standard MPP_j_ for different grades of farmland, from high to low, is as follows: The representative yield potential standard per unit of farmland in first-class areas is calculated to be 10.28 t. For second-class areas, the representative yield potential standard per unit of farmland is calculated to be 9.94 t; The representative yield potential standard for unit farmland in third-class areas is calculated to be 9.31 t. In fourth-class areas, it is 8.91 t, and in fifth-class areas, it is 7.95 t, as illustrated in Fig 6.

### 5.3. Economic value assessment results

Based on the standard unit yield of farmland across different quality levels, the economic value per unit area was calculated using the soil potential estimation method. The results are as follows: The economic value per unit area of first-grade farmland is 3,291.13 yuan/hectare. The economic value per unit area of second-grade farmland is 3,181.62 yuan/hectare. The economic value per unit area of third-grade farmland is 2,983.61 yuan/hectare. The economic value per unit area of fourth-grade farmland is 2,851.69 yuan/hectare. The economic value per unit area of fifth-grade farmland is 2,544.27 yuan/hectare.

Combining these values with the total area of farmland across different quality levels, the economic total value of farmland in Hefei, Anhui Province, in 2021 was calculated to be 1.363 billion yuan. The breakdown by farmland quality level is as follows: The economic value of first-grade farmland is 81 million yuan. The economic value of second-grade farmland is 158 million yuan. The economic value of third-grade farmland is 273 million yuan. The economic value of fourth-grade farmland is 444 million yuan. The economic value of fifth-grade farmland is 407 million yuan. This pattern highlights an important implication: although first-grade farmland shows the highest productivity and economic return per hectare, it is the extensive area of lower-grade farmland that predominantly drives the overall economic value, which is critical for regional agricultural policy and land-use planning. The relatively high total value of low-grade farmland is primarily driven by its large area. Despite having the lowest unit productivity and economic value per hectare, fifth-grade farmland constitutes approximately 33% of the total farmland area in Hefei. This large coverage substantially contributes to its share of the overall economic value of farmland.

Analyzing the results by administrative regions, among all the prefecture-level cities in Hefei, those with farmland economic values exceeding 100 million yuan include Chaohu, Feidong, Feixi, and Lujiang. Regions with economic values exceeding 10 million yuan but less than 100 million yuan include Shushan and Yaohai. Regions with economic values below 10 million yuan include Luyang and Baohe. The detailed breakdown of unit and total economic values by farmland grade is presented in Table 6.

**Table 6:** Statistics on the classification of the economic value of farmland.

	First class	Second class	Third class	Fourth class	Fifth class	All
The economic value of farmland units at all levels(Yuan/ hectare)	3291.13	3181.62	2983.61	2851.69	2544.27	——
Economic total value of farmland(Yuan)	81265207.27	157968737.46	272950190.35	443762887.66	407168407.60	1363115430.35

All economic values are uniformly reported in yuan/hectare for unit values and yuan for totals.

## 6. Discussion

### 6.1. Discussion of research findings

In this study, the final farmland economic valuation results for Hefei City in 2021 reveal that the proportion of economic value contributed by different quality grades of farmland is influenced by their respective area distributions. Specifically, first-grade farmland contributes about 7.6% of the total economic value, second-grade farmland about 11.6%, third-grade about 20%, fourth-grade about 33%, and fifth-grade about 30%. Medium- and low-grade farmland, which occupies the largest area, accounts for over 60% of the economic total value. In contrast, medium- and high-grade farmland, with smaller area proportions, contributes approximately 30% of the economic total value. High-grade farmland, with the smallest area, contributes less than 10% of the economic total value. This indicates that both area and productivity potential jointly determine farmland value.

However, the area data for different quality grades exhibit greater variability compared to productivity potential results. Therefore, the economic value is more significantly affected by area distribution. This finding suggests that spatial structure and farmland layout play a more decisive role than marginal differences in productivity potential. Hence, improving farmland layout and optimizing land use patterns could yield greater economic benefits than minor improvements in soil productivity alone.

The variability in farmland quality grade data is inevitably influenced by the classification method used for the Comprehensive Farmland Quality Index (CFQI) [1]. This study employs the natural breaks method for classification instead of the equal-interval method specified in the farmland Quality Grading standard [51]. Such adjustment reduces differences in area proportions among quality grades and enhances the scientific validity and rationality of the valuation results.

### 6.2. Comparison with other studies

#### 6.2.1. Smaller variations in farmland economic value in this study.

Comparisons with other studies show that the unit economic values of farmland across county-level administrative units in Hefei City differ slightly from those in this study [52]. The unit economic values in this study are generally lower. In contrast, values reported in other studies tend to be significantly higher. For example, some scholars used the income capitalization method in Anhui Province and found over 20-fold differences between counties, contrasting with the approximately 3,000 yuan/hectare differences observed here [53]. This discrepancy may stem from differences in valuation perspectives and methodological approaches.

This study calculates the economic value based on farmland quality grades. It considers the distribution of different quality grades within each county and sums the standard economic values of each grade to obtain the total economic value. The total value is then divided by the county area to derive the unit economic value. This approach inherently homogenizes the economic value within each county. In contrast, other studies use the income capitalization method, which incorporates annual yields and market values. As a result, those estimates are more susceptible to regional market conditions and labor input-output variations, leading to larger discrepancies in unit economic values [11,54].

Another reason may be that this study does not consider capitalization rates and only calculates the economic value for a single year. Other studies, however, capitalize income over an infinite period, which naturally produces larger overall values. Despite the differences in absolute values, the smaller intra-county variations in economic values in this study demonstrate the feasibility of the proposed methodology. A summary comparison is provided in S1 Table (Supplementary Material), which visually highlights these differences.

These differences also reflect the underlying differences in methodological assumptions. Income capitalization methods, as applied by researchers such as Chen et al. (2023) and Li et al. (2018), assume that farmland value stems primarily from its expected economic returns over time, typically estimated through future yield projections and market prices [9,54]. In contrast, this study adopts a natural attribute-based approach, focusing on soil fertility, landform, and climatic potential as objective indicators of value. While capitalization approaches tend to exaggerate spatial disparities due to their sensitivity to local market volatility and labor inputs, our method reduces such volatility by grounding value in stable ecological parameters. This contrast highlights the complementary yet distinct insights offered by each method and highlights the need for multiple valuation perspectives in farmland management.

#### 6.2.2. Valuing farmland based on natural attributes provides a more targeted research perspective.

Most existing studies on farmland economic valuation focus on economic attributes, such as market prices, output benefits, and production costs, while neglecting the influence of natural attributes on economic value. This study takes a different approach by calculating farmland economic value based on natural attributes. It constructs a Comprehensive Farmland Quality Index (CFQI) and classifies farmland areas by quality grades. Additionally, the CFQI is used to adjust farmland productivity potential, yielding unit-area yields for different quality grades. By examining farmland value from the perspective of natural attributes, this study achieves more precise grading and reveals the economic value differences among quality grades. This provides a more targeted and insightful approach to farmland economic valuation.

This natural attribute-based valuation framework exhibits several key advantages. First, it is highly objective because it relies on physical environmental data such as soil, topography, and climate. These factors are measurable and not subject to human preference or market bias. Second, the method is replicable since it uses standardized models—such as the Thornthwaite-Mather model—and quantitative indicators. This ensures consistent application across different regions. Third, by anchoring farmland value in its ecological foundation—such as water availability and soil productivity—it provides a science-based rationale for sustainable land-use planning. Together, these strengths make the natural attribute-based framework particularly valuable for ecological compensation policy, conservation planning, and long-term farmland protection.

#### 6.2.3. Using grid scale as the valuation unit enhances precision.

Existing farmland valuation studies primarily operate at macro and meso scales, with administrative regions and land parcels as the main evaluation units. In contrast, this study adopts a micro-scale approach and uses grid cells as the valuation units. A total of 20,439 evaluation units were established for the entire study area.

The attribute data of farmland quality grade indicators are processed through grid-based calculations to obtain the CFQI for each evaluation unit. These CFQI values were then used to classify farmland in Hefei City into five quality grades. The CFQI of each evaluation unit is multiplied by the farmland productivity potential to derive the adjusted productivity potential.

Using grid-scale data acquisition and processing allows farmland value attributes to be expressed and computed in a spatially explicit manner. This approach leads to more precise and spatially detailed economic valuation results.

#### 6.2.4. Constructing value calculation indicators based on natural ecological characteristics enhances universality.

Existing farmland economic valuation methods—such as market-based, cost-based, and substitution approaches—are widely used. However, they are significantly influenced by regional economic development levels, land market maturity, and policy environments. These influences limit their applicability and accuracy across regions.

This study employs the Thornthwaite Memorial Model, which uses annual actual evapotranspiration and precipitation as climatic variables. These variables are used to characterize farmland productivity and derive initial economic values. Because evapotranspiration and precipitation are natural factors, they are not affected by market price fluctuations or regional economic differences. This feature makes the method widely applicable both regionally and globally.

Existing farmland quality grading systems often incorporate natural, economic, and social factors. In contrast, the CFQI in this study is constructed solely on natural environmental factors. This design avoids the influence of regional economic disparities, policy environments, market interferences, and social conditions. As a result, it enhances the applicability, objectivity, and universality of the evaluation indicators.

Moreover, incorporating grid-scale approaches aligns with recent ecosystem service valuation practices. Wu et al. (2024) and Vidal et al. (2021) both leveraged grid-based models to capture spatial heterogeneity, supporting robust and transferable assessments across regions [22,55]. This convergence highlights the global relevance and replicability of grid-based natural attribute frameworks for farmland valuation.

#### 6.2.5. Introducing CFQI to adjust unit farmland economic value improves precision.

Existing studies use the CFQI to calculate farmland area by quality grades but do not apply it to adjust productivity potential. In this study, the CFQI is used not only to classify farmland areas but also to adjust productivity potential. This adjustment produces more accurate unit-area economic values.

By incorporating the CFQI, the relationship between farmland quality and productivity is more precisely quantified [24]. This ensures that the economic valuation aligns more closely with real-world production conditions. As a result, the overall accuracy of the valuation results is improved.

### 6.3. Limitations and future research directions

#### 6.3.1. Expanding indicators for site conditions, locational factors, and ecological factors to form a more comprehensive evaluation framework.

Due to data limitations, the current CFQI indicator system only includes basic indicators for site conditions, locational factors, and ecological factors. This may not fully capture the diversity and complexity of factors influencing farmland quality [56].

Future studies could integrate additional data sources, such as nighttime light data (from VIIRS/DMSP), road network datasets (from OpenStreetMap or national transportation bureaus), and crop suitability maps (from FAO or local agricultural surveys), to support proposed new indicators. These datasets would allow for the inclusion of variables capturing socioeconomic activity, accessibility, and environmental suitability.

Future research could introduce indicators such as “light conditions” and “crop adaptability” under site conditions, “distance to main roads” and “irrigation facility coverage” under locational factors, and “surrounding land use types” and “agricultural product value provision” under ecological factors. Such additions would create a more comprehensive evaluation framework, enhancing both the flexibility and precision of farmland quality assessment.

#### 6.3.2. Overcoming regional meteorological data limitations to develop a universal farmland productivity potential assessment method.

The Thornthwaite Memorial Model used in this study relies on regional temperature, precipitation, and evapotranspiration data. These dependencies may introduce biases in areas with unique climatic conditions, such as high-latitude, arid, tropical, or high-altitude regions, as well as those with strong seasonal precipitation variability [57].

Future research could also compare results with global NPP datasets, such as MODIS NPP products. This comparison would provide an external validation check and help identify potential model deviations across climatic zones.

To develop a globally applicable farmland productivity potential assessment method, future research should explore approaches based on stable variables such as inherent farmland attributes and crop characteristics. This would reduce reliance on meteorological data and help overcome regional limitations.

#### 6.3.3. Discounting one-year values to accurately reflect farmland economic value.

This study uses 2021 economic values to represent the total farmland economic value in Hefei City, based on the assumption that one-year values reflect the overall economic value. However, farmland provides continuous agricultural outputs and long-term economic benefits. Therefore, infinite-period valuation offers a more accurate reflection of its sustained value. One-year values are easily affected by short-term market fluctuations. These fluctuations may lead to potential overestimation or underestimation of farmland value.

A simple sensitivity test could apply a discount model (e.g., PV = V₁/ (1 + r)), with r approximately 3–5%, to illustrate how present values change under different discount rates. Future research could apply such discounting to convert one-year values into infinite-period equivalents. This would incorporate the time value and uncertainty of future earnings, providing a more stable and accurate representation of farmland economic value.

#### 6.3.4. Acknowledging limitations and the need for validation.

This study has several limitations that should be acknowledged. First, the indicator system used in the CFQI framework primarily covers a basic set of site, location, and ecological conditions. While these provide a solid foundation, they do not encompass all factors influencing farmland quality, such as detailed soil nutrient dynamics, climate extremes, or socio-economic interactions.

Second, the Thornthwaite-Mather model used for estimating climatic productivity potential is subject to regional limitations. These limitations are especially evident in areas with atypical precipitation patterns or high-altitude conditions. Therefore, caution is warranted when extending this model beyond regions with similar climatic characteristics.

Third, this study is based on data from a single year (2021). This temporal limitation may not fully capture interannual variations in climate, yield, and prices. Future work should incorporate multi-year datasets or apply economic discounting methods to improve valuation stability over time.

Additionally, although the modeled farmland productivity potential aligns with expected spatial gradients, this study did not conduct direct validation using observed crop yields or remote-sensing-derived NPP datasets. Future research should integrate cross-validation with satellite-based NPP products, such as MODIS NPP, along with field-observed yield records. This approach would help assess model performance and enhance the reliability of the results.

### 6.4. Research significance

#### 6.4.1. Integrating natural attributes to deepen farmland economic value theory and inform policy.

Existing farmland economic valuation studies emphasize economic attributes while neglecting the intrinsic value of farmland as a natural resource. By highlighting the foundational role of natural attributes, this study refines the understanding of farmland value formation. It also contributes to the broader theory of economic valuation. In addition, the study underscores the importance of incorporating natural conditions into farmland management and planning frameworks.

Under the context of scarce farmland resources, this natural attribute-based perspective supports the formulation of resource-efficient and sustainable policies. For instance, municipal planners could prioritize the protection of Class I farmland, which accounts for only about 5% of area but contributes roughly 6% of total value. This targeted approach helps guide both the protection and the rational utilization of farmland resources. It also provides a theoretical basis for government decision-making on land resource allocation and enhances land-use efficiency.

#### 6.4.2. Valuing farmland based on natural attributes offers new perspectives and methods for addressing market failures.

Under existing market mechanisms, the natural attributes of farmland—characterized by externalities and non-market features—are often undervalued. This undervaluation leads to resource misallocation and overexploitation. By quantifying the contribution of natural conditions to economic activities, this study provides a scientific basis for farmland resource management and protection.

The natural attribute-based valuation framework supports the setting of farmland protection targets. It also helps optimize land use structures and promote green development policies. Additionally, this framework offers a pathway for internalizing externalities through ecological compensation and resource taxation. Such measures encourage sustainable land management practices and mitigate the negative impacts of market failures.

#### 6.4.3. Scientifically quantifying farmland natural attributes enhances applicability, stability, replicability, and scalability.

The natural attribute-based economic valuation method integrates natural conditions into the core framework of farmland economic assessment. By scientifically quantifying natural attributes, the method addresses regional disparities. It also enhances the applicability and stability of the valuation results. The method relies on objective, quantifiable data and standardized calculation steps. This ensures high replicability and scalability, providing a unified scientific tool for farmland resource management and policy-making across regions.

#### 6.4.4. Broadening the impact through global contextualization and future research pathways.

This study contributes to the evolving field of ecosystem service valuation by embedding farmland economic assessment within a natural attribute framework. This approach enhances objectivity, reproducibility, and ecological relevance. These are key advantages over conventional market-based valuation methods.

To expand the broader applicability of this work, future research should integrate global ecosystem service benchmarks, such as the valuation coefficients developed by Costanza or the TEEB framework [58]. These references provide standardized economic values for multiple land cover types. They also enable meaningful cross-regional comparisons.

Additionally, future research could explore dynamic valuation models that incorporate inter-annual variations in productivity, climatic change effects, and long-term land use transitions. Such extensions would align farmland valuation more closely with sustainability science. They would also improve its practical utility for global land management and policy design.

## 7. Conclusion

This study developed an integrated framework for assessing the economic value of farmland by combining the comprehensive fertility index method, the Thornthwaite–Mather model, and the soil potential estimation method. Centered on farmland quality grading and production potential assessment, the framework provides an objective and reproducible approach to quantify the economic value of farmland based on its natural attributes.

The empirical results from Hefei, China, show clear spatial differentiation in farmland value across quality grades. In 2021, Hefei’s farmland covered approximately 481,500 ha. The highest-grade farmland accounted for about **5%** of the total area, while the lowest-grade farmland accounted for roughly 33%. Although higher-grade farmland exhibited greater productivity and unit economic value, the extensive area of lower-grade farmland contributed substantially to the city’s total farmland value, which was estimated at 1.363 billion CNY.

These findings reveal that farmland area and productivity jointly determine overall economic value. The results also highlight the scientific validity of the natural-attribute–based valuation framework. By grounding value in ecological and biophysical indicators, this approach reduces interference from market and policy fluctuations and improves consistency across regions.

This research advances farmland valuation theory by integrating natural attributes into the assessment of economic value. It provides a unified and transferable tool for farmland protection, sustainable land-use planning, and ecological compensation policy. Future studies could extend this framework by incorporating long-term time-series data and discounting methods to capture interannual variability and the dynamic value of farmland resources.

## Supporting information

S1 Appendix Model parameter details and validation notes. This appendix presents an example of pairwise comparison matrix entries used in the Analytic Hierarchy Process (AHP). The table illustrates how different criteria—Soil Fertility (SF), Topography (T), Irrigation Condition (IC), and Accessibility (A)—were comparatively evaluated to derive weights for the farmland quality assessment model.
(DOCX)

S1 Table Farmland productivity classification thresholds by grade. This table compares the variation in farmland unit economic value under two different assessment approaches. The income capitalization method (Hu et al., 2014) shows large inter-county disparities in Anhui Province, while this study’s natural-attribute-based method results in a significantly narrower range in Hefei City, thereby illustrating its robustness and consistency.
(DOCX)
