# Supplementary material for: Economic valuation of farmland using natural-attribute–based indicators: A case study of Hefei, China
Source: PLoS One. 2025 Dec 30;20(12):e0337934. doi: 10.1371/journal.pone.0337934 (PMC12752984; doi:10.1371/journal.pone.0337934)
Supplement: S1 Appendix — This appendix presents an example of pairwise comparison matrix entries used in the Analytic Hierarchy Process (AHP). The table illustrates how different criteria—Soil Fertility (SF), Topography (T), Irrigation Condition (IC), and Accessibility (A)—were comparatively evaluated to derive weights for the farmland quality assessment model. (DOCX) [file pone.0337934.s001.docx]

Appendix A.

S1 Appendix. Example of Pairwise Comparison Matrix Entries

| Criteria | SF | T | IC | A |
| --- | --- | --- | --- | --- |
| Soil fertility (SF) | 1 | 5 | 3 | 7 |
| Topography (T) | 1/5 | 1 | 1/2 | 3 |
| Irrigation condition (IC) | 1/3 | 2 | 1 | 5 |
| Accessibility (A) | 1/7 | 1/3 | 1/5 | 1 |
