# Supplementary material for: Economic valuation of farmland using natural-attribute–based indicators: A case study of Hefei, China
Source: PLoS One. 2025 Dec 30;20(12):e0337934. doi: 10.1371/journal.pone.0337934 (PMC12752984; doi:10.1371/journal.pone.0337934)
Supplement: S1 Table — This table compares the variation in farmland unit economic value under two different assessment approaches. The income capitalization method (Hu et al., 2014) shows large inter-county disparities in Anhui Province, while this study’s natural-attribute-based method results in a significantly narrower range in Hefei City, thereby illustrating its robustness and consistency. (DOCX) [file pone.0337934.s002.docx]

Table S1. Comparison of farmland unit economic value variation

| Method | Study area | Range of differences |
| --- | --- | --- |
| Income capitalization (Hu et al., 2014) | Anhui Province | >20-fold between counties |
| This study (natural attribute) | Hefei City | ~3,000 yuan·hm^-2^ |
